# Ultrasound of the Normal Brachial Plexus

**DOI:** 10.5334/jbr-btr.1418

**Published:** 2017-12-16

**Authors:** Paolo Simoni, Merhan Ghassemi, Vinh Dat-Minh Le, Grammatina Boitsios

**Affiliations:** 1 Reine Fabiola Children’s University Hospital, Université Libre de Bruxelles, BE

**Keywords:** Brachial plexus, Ultrasound anatomy, Thoracic outlet syndrome, Tumor invasion, Trauma

## Abstract

Ultrasound (US) allows a reliable examination of the brachial plexus except for the spinal nerve roots, located deep in the neuro-foramina, beyond the shadowing of the transverse processes of the vertebral bodies. All the other fascicles of the brachial plexus can be mapped by US from the roots of the spinal cervical nerves, from C5 to T1 to the branches at level of the axillary region.

US can be considered as an alternative to Magnetic Resonance Imaging (MRI) when MRI is contraindicated, not readily available or in case of claustrophobia. US can be used for the assessment of the brachial plexus in case of trauma, tumours and fibrosis induced by radiation oncology treatments. US is also a valuable tool to perform imaging-guided blocks of the brachial plexus. A prerequisite for a reliable US examination of the brachial plexus is knowledge of its complex anatomy. The operator is also required to learn the appropriate US views in order to have an optimal depiction of the brachial plexus, especially the areas where the bone structure’s interposition makes the visualisation of the brachial plexus more arduous.

The aim of this review is to provide the reader with the basics principles of the US normal anatomy and technique for a reliable mapping of the brachial plexus.

## Introduction

Ultrasound (US) is a largely available, cost-effective and innocuous technique to assess the brachial plexus. US can be considered as an alternative to Magnetic Resonance Imaging (MRI) in all clinical settings in which MRI is contraindicated, not readily available or in case of claustrophobia [1]. US can be used for the assessment of the brachial plexus in case of trauma, tumours and fibrosis induced by radiation oncology treatments [1,2]. US is also used to perform imaging-guided blocks of the brachial plexus at various levels [3].

## Brachial Plexus Anatomy and Mnemonics

A clear understanding of the brachial plexus anatomy is a prerequisite for a fast and reliable ultrasound exploration [1,2,3,4,5,6,7].

The brachial plexus arises from the ventral rami of the spinal nerves from C5 to T1. The ventral rami of the spinal nerves create a complex neural network, which supplies the sensory, motor and autonomic innervation of the shoulder girdle and upper limb.

The dorsal rami of the spinal do not contribute to the brachial plexus formation but they supply the muscles and the skin of the paravertebral area of the neck and of the posterior part of the scalp (occipital, sub-occipital and auricular nerves) [6]. Unlike the other levels of the spine, cervical spine roots emerge above the pedicles with the same name (e.g. the C5 root emerges from the neuro-foramen between the C4 and C5 vertebral bodies) [8].

The brachial plexus consists of five roots (from C5 to T1), three trunks (superior, middle and lower), six divisions (three anterior and three posterior), three cords (medial, posterior, lateral) and five branches (musculocutaneous, axillary, radial, median and ulnar nerves) [2,5,6].

One possible mnemonic to remember this complex anatomy is the following sentence: **R**emember **T**o **D**rink a **C**old **B**eer (**R**ami, **T**runks, **D**ivision, **C**ords, **B**ranches). The number of these components can be easily memorised by the symmetric number **53635** (**5** rami, **3** trunks, **6** divisions, **3** cords, **5** branches).

The mnemonic **MARMU** can be used to easily remember the name of the branches of the brachial plexus in the correct order (**M**usculocutaneous, **A**xillary, **R**adial, **M**edian and **U**lnar nerves).

**Figure 1:**
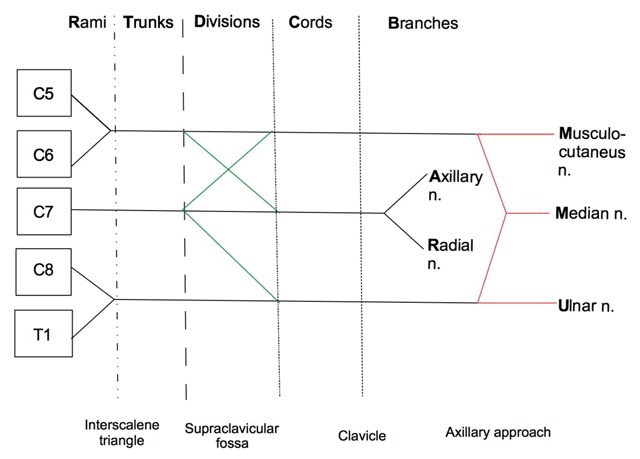
**Scheme representing the brachial plexus.** A drawing can easily be obtained by representing the vertebral bodies vertically from C5 to T1 and the different components horizontally (for the components one can use the mnemonic: **R**un **T**o **D**rink a **C**old **B**eer). Three “Y” can be traced from the vertebral bodies. The “Y” can be traced the intermediate with the fork pointing distally (black in Figure 1). An “X” and an inverse slash can to be added at level of the division as shown in the scheme (in green). Finally, a S (in red in the figure) can be drawn to represent three of the 5 branches; the intermediate “Y” provides the other two branches. In the lower line the corresponding main anatomic landmarks are represented. This is the basic scheme, but other smaller branches (usually not visible or not investigated by US) can be added.

A scheme of the brachial plexus can be quickly drawn on a piece of paper as illustrated in Figure 1. The following key landmarks can help in placing the above-mentioned scheme in the real anatomy when performing a US mapping of the brachial plexus [1,2,5,6]:

- The cervical vertebral bodies (for rami);
- Scalene muscles and subclavian artery (for rami and trunks);
- The first rib outline and the subclavian artery (for divisions);
- Clavicle, subclavian and axillary vessel and pectoralis minor muscle (for cords);
- Brachial vessels, humerus and muscle around the shoulder (for branches).

## Technical Considerations

Because the brachial components are small in size and they become superficial only beyond the inter-scalene triangle, the use of linear probes of 5–12 MHz has been recommended as a the best compromise to obtain an optimal penetration and adequate definition of the more superficial fascicles (e.g. cords) [1,2]. In addition most 5–12 MHz probes are larger than those with a higher frequency, thus providing a large field of view. However, the operator may experience some difficulties when using these large probes in short-neck individuals or in toddlers.

Higher frequency linear probe (e.g. 18 MHz probe) can be employed in thin individuals, in children or to better approach superficial regions such as supraclavicular fossa and around the costo-clavicular outlet because most suppliers provide high-frequency linear probes in an a small size compared to the 5–12 MHz probes.

The use of the Doppler during US examination of the brachial plexus is particularly suitable to better recognise the above-mentioned vascular landmarks adjacent to nerve fascicles [1,2,5], although it can deteriorate the B-Mode images quality. Doppler images are also used as a guidance to perform blocks of brachial plexus by various approaches [1,2].

Each US device should be carefully set on some volunteers to optimise the image quality, because the other standard settings (e.g. thyroid or musculoskeletal) may turn out to provide suboptimal images [4].

One must bear in mind, especially when dealing with patients with a suspected traumatic injury of the brachial plexus, that ultrasound is not able to image the spinal nerve roots. These structures are deeply located in the spinal canal, beyond the acoustic shadowing of the vertebral tubercles [2].

## US Examination Technique

### US approaches to the brachial plexus

Two general approaches have been suggested to map the brachial plexus by ultrasound for diagnostic purposes: the first using axial and the second using oblique sagittal views [1,2]. The approach using axial views proposed by Martinoli and al. [6] is probably easier to learn for beginners. Axial views allow for ready identification of the spinal roots/rami and the division of the brachial plexus. The sagittal oblique views described by Demondion and colleagues [2] have the advantage of reproducing the views of the brachial plexus of the most common magnetic resonance imaging protocols [2]. In practice, learning and practicing both approaches can be useful in depicting the fascicles of the brachial plexus in different planes.

### Roots and their ventral rami

The brachial plexus examination begins with the visualisation of the cervical roots from C5 to T1, which are the origin of the ventral rami of the brachial plexus [1,2].

**Figure 2:**
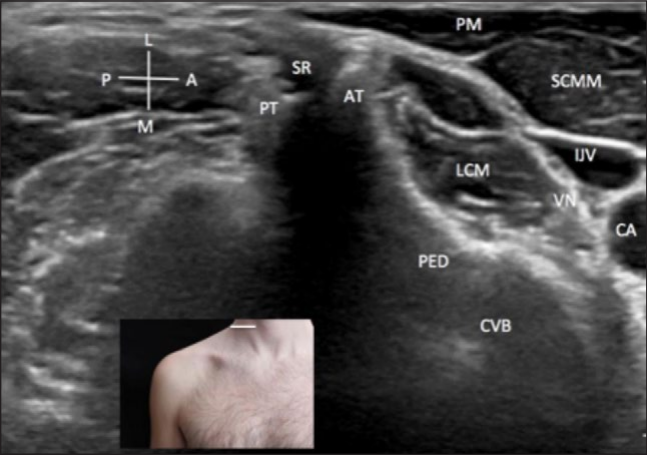
**Roots/rami of the brachial plexus – axial view.** A: anterior. AT: anterior tubercle. CA: carotid artery. CVB: cervical vertebral body. IJV: internal jugular vein. L: lateral. LCM: longus colli muscle. M: medial. P: posterior. PED: pedicle. PM: platisma muscle. PT: posterior tubercle. SCMM: scalenus medium and posterior muscle. SR: spinal root. VN: vagus nerve. The probe position is indicated by the white bar in the insert.

**Figure 3:**
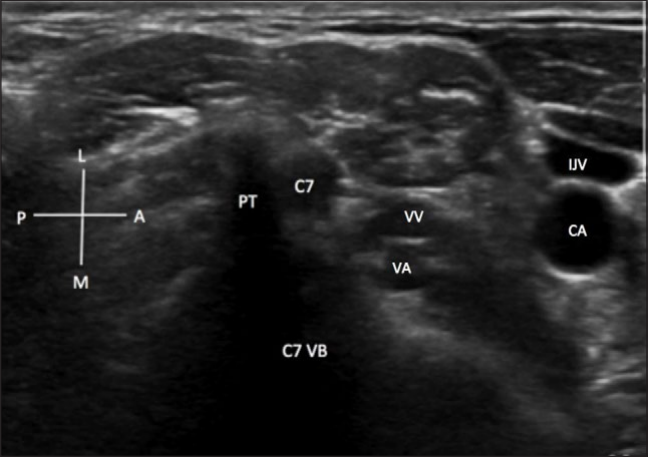
**C7 spinal root – axial view.** Note this absence of the anterior tubercle at this level: A: anterior. C7VA: C7 vertebral body; CA: carotid artery. CVB: cervical vertebral body. IJV: internal jugular vein. M: medial. L: lateral. LPVA: vertebral artery. P: Posterior. VA: vertebral artery. VV: vertebral vein.

The patient is positioned supine with the head turned opposite to the side to be explored (Figure 2). In the axial view, the probe is initially placed on the thyroid, and the probe is therefore shifted slightly laterally to image the common carotid artery, the internal jugular vein and the vagus nerve into the carotid sheet (Figure 2). These neurovascular structures are located between the sternocleidomastoid muscles, anteriorly, and the longus colli muscle posteriorly. The bone shadowing of the lateral aspect of the vertebral body can be seen clearly deeply these structures if the depth of the image is well set (Figure 2). The emergence of the roots and ventral rami can be visualized just posterior to the longus colli muscle, between the anterior and posterior vertebral tubercles of each transverse process. The roots and their ventral rami are mono-fascicular homogeneous hypo-echoic structures in the vertebral vessels situated anteriorly and visible on Doppler images between the transverse processes when moving the probe cranially and caudally [6]. Because the C7 vertebra has no anterior tubercle (Figure 3), this is landmark is used in the axial plan to determine the of the C7 root and to correctly name the other roots by moving the probe upward or downward [1]. The C8 and T1 can be difficult to see in the axial view because of the sterno-clavicular joint.

**Figure 4:**
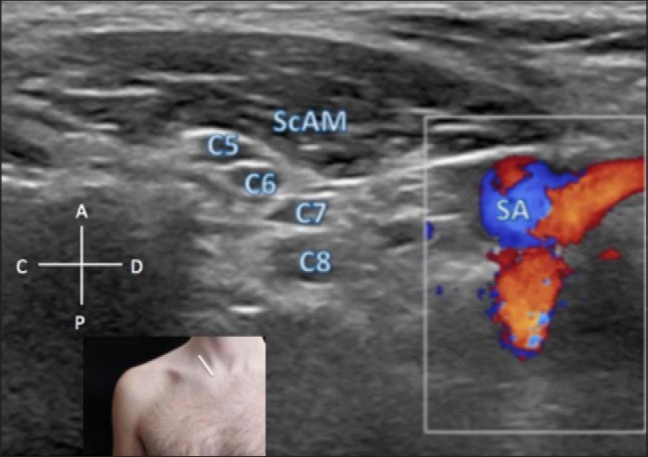
**Roots/rami of the brachial plexus – sagittal oblique view.** A: anterior. C: cranial. C4: C4 spinal root. C5: C5 spinal root. C6: C6 spinal root. C7: C7 spinal root. C8: C8 spinal root. D: distal. M: medial P: posterior. SA: subclavian artery. ScAM: scalenus anterioris muscle. The probe position is indicated by the white bar in the insert.

In the sagittal oblique plane the probe is placed on the inferior third of the sternocleidomastoid muscle, perpendicular to the main axis of the supra-clavicular vessels (Figure 4). In this view, the emerging roots are seen as ovoid hypo-echoic images interposed between the anterior and middle scaleni muscles. Moving the probe just a few millimetres posteriorly, keeping the same oblique incidence of the probe, the deep cervical artery (a branch of the costo-vertebral artery) can be seen running posteriorly between the C7 and C8 roots, thus providing a landmark to recognise these two roots in the sagittal oblique plane [2].

The C8 root is located just posterior and slightly superior to the subclavian artery.

Even in sagittal oblique planes the C8 and T1 roots are not visible in all individuals [1,2], because of the deep location of neuro-foramen between the T1 and T2 vertebrae behind the clavicle and the first axial rib. An axial oblique view at 45 degrees following the course of the T1 root can help to make visualisation simpler in some cases [1].

### Trunks

**Figure 5:**
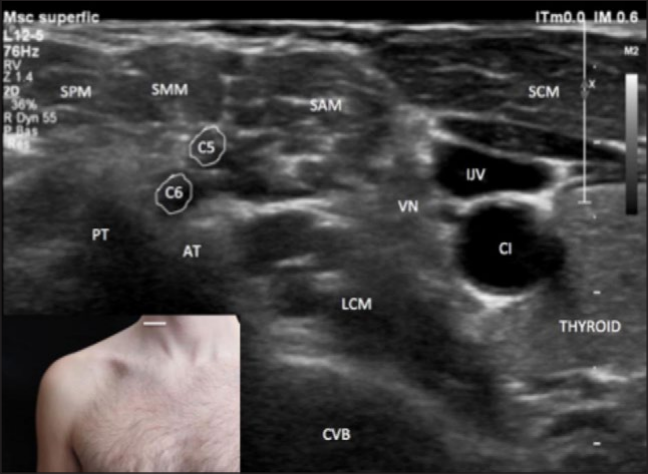
**Trunks of the brachial plexus formation – axial view.** AT: anterior tubercle. C5: C5 spinal root. C6: C6 spinal root. CVB: cervical vertebral body. IJV: internal jugular vein. LCM: longus colli muscle. SAM: scalene anterioris muscle. SMM: scalenus medius muscle. SPM: scalenus posterioris muscle. SCM: sternocleidomastoid muscle. PT: posterior tubercle. VN: vagus nerve. The probe position is indicated by the white bar in the insert.

**Figure 6:**
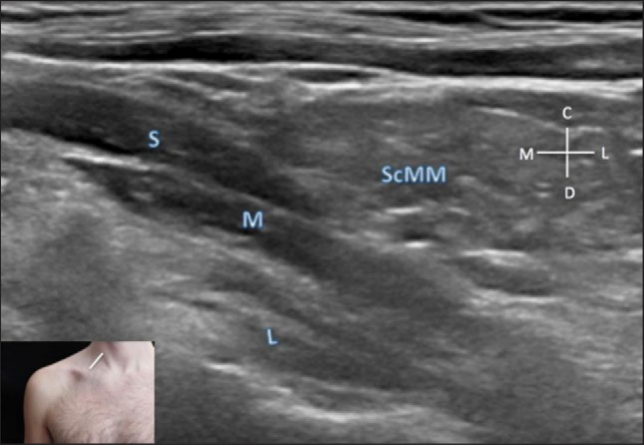
**Trunks of the brachial plexus – coronal view.** C: cranial. D: distal. L: lower trunk. L (white): lateral. M (white): medial. M: middle trunk. S: superior trunk. ScMM: scalenus medius muscle. The probe position is indicated by the white bar in the insert.

In the axial plane, the three trunks of the brachial plexus can be seen between the anterior and middle scalene muscle, always in the inter-scalene triangle area, in continuity with the spinal rami (Figure 5). The presence of a sufficient amount of the hyper-echoic fat tissue in the inter-scalene triangle help distinguish the rami and the corresponding trunks from the nearby muscular fascicles (Figure 5). The operator must bear in mind that the cranial roots are more laterally located in the inter-scalene triangle on the axial view (i.e. the C5 ramus is located superficial to the C6 ramus) (Figure 5). Sweeping the probe down along the vertical axis, the axial allows for observation of the formation of the upper trunk (C5 + C6), the middle trunk (C7 alone) and the inferior trunk (C8 + T1) from the rami at the outer part of the inter-scalene triangle (6). These three trunks can be easily shown in the inter-scalene triangle (Figure 6). The trunks appear as a set of three slightly curved hypo-echoic fascicles located superior to the subclavian artery.

### Divisions

The six divisions of the brachial plexus can be found at the lateral third of the supraclavicular fossa by a sagittal oblique slice, perpendicular to the main axis of the subclavian artery (Figure 6).

The use of a high-frequency liner probe with small width (e.g. a 15 MHz “golf” probe or a 18 MHz linear probe) enables an easy approach to this hollow region with a better spatial resolution.

A cluster of hypo-echoic fascicles corresponding to the six divisions of the brachial plexus is located just behind the subclavian artery. In this view, both artery and divisions of the brachial plexus rely on an arch made of the shadowing of the superior aspect of the first rib. The cluster of the six divisions is approximately double in size compared to the adjacent artery [2].

### Cords

**Figure 7:**
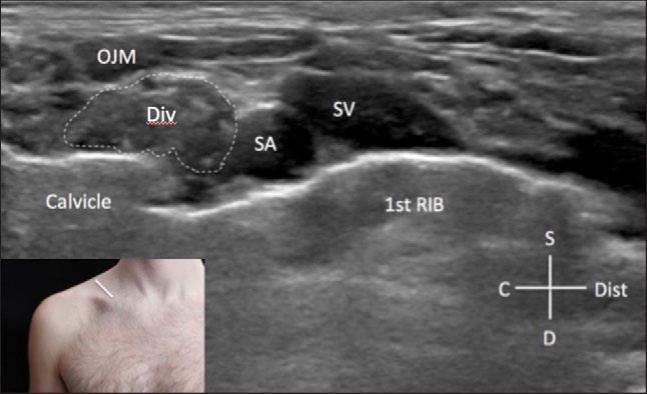
**Divisions of the brachial plexus.** C: cranial. D: deep. Div: divisions of the brachial plexus. Dis: distal. OJM: omohyoid muscle. SA: subclavian artery. SV: subclavian vein. S: superficial. The probe position is indicated by the white bar in the insert.

**Figure 8:**
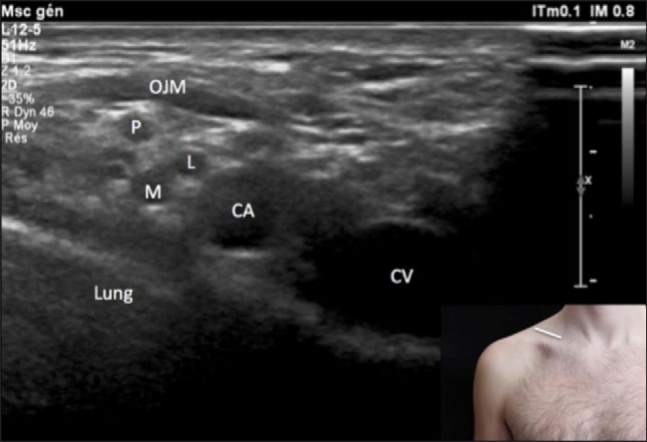
**Cords of the brachial plexus – supraclavicular view.** CA: subclavian artery. CV: subclavian vein. M: medial cord. L: lateral cord. OJM: omohyoid muscle. P: posterior cord. The probe position is indicated by the white bar in the insert.

The formation of the posterior, medial and lateral cords occurs just at the costoclavicular space. Since the space behind the clavicle is difficult to visualise at US because of the bone shadowing, two main views are possible: the supraclavicular (Figure 7) and infra-clavicular (Figure 8) [2]. The former is obtained with the probe placed at the middle third of the clavicle just above its superior margin (Figure 7), the latter with the probe just (Figure 8). The probe is always oriented perpendicular to the main axis of the subclavian artery (Figures 7 and 8).

In both views the cords of the brachial plexus form a triangular hypo-echoic image (also called “Phrygian cap” appearance) located just above the subclavian artery: the posterior cord is located at the upper part of the triangle, the lateral cord is visible at the anterior and inferior part of the triangle, while posterior and inferior part of the triangle corresponds to the posterior cord [2] (Figures 7 and 8).

**Figure 9:**
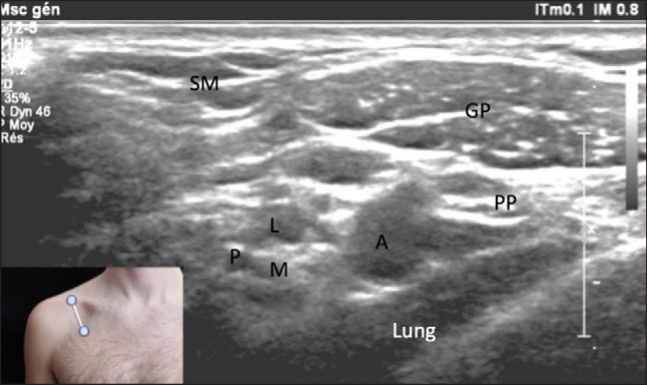
**Cords of the brachial plexus – infraclavicular view.** CA: subclavian artery. CV: subclavian vein. M: medial cord. L: lateral cord. P: posterior cord. GP: pectoral major muscle. PP: pectoralis minor muscle. The probe position is indicated by the white bar in the insert.

In the infra-clavicular view and the cords of the brachial plexus keep the same spatial pattern and the same anatomical relationship with the subclavian artery but they are visible deep to the subclavius muscle more proximally (Figure 8) and under the pectorialis minor muscle more distally when the probe is shifted perpendicular toward the delto-pectoral groove, following the axillary artery (Figure 9).

### Branches

**Figure 10:**
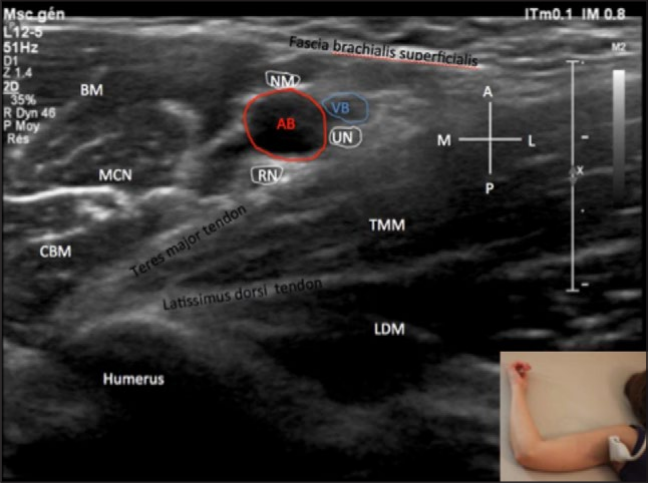
**Branches of the brachial plexus – branches other than the axillary nerve – axillary approach.** A: anterior. AB: Brachialis artery. BM: Brachialis muscle. CBM: coracobrachialis muscle. L: lateral. LDM: Latississimus dorsi muscle. NM: Median nerve. M: medial. MCN: musculocutaneus nerve. P: posterior. RN: radial nerve. TMM: UN: ulnar nerve. VB: brachialis vein. The probe position is indicated by the white bar in the insert.

The three brachial plexus cords divide in five terminal branches after running underneath the pectorals minor muscle. These branches are visible at the root of the upper limb. In order to map at the ultrasound these branches, the patient must to be positioned supine with the arm abducted to 90 degrees, the forearm supinated and flexed at 90 degrees (Figure 10) [7]. In this position the US probe can be placed in the sagittal plane at the axilla perpendicular to the skin at indentation between the biceps muscle and pectorals major muscle as shown in Figure 9. From this position the probe can be moved distally toward the elbow. The main landmarks to map the branches of the brachial plexus at the junction between the axilla and the upper arm are the axillary vessels. The median nerve is located anterior to the artery, while the ulnar nerve is visualised between the axillary (Figure 10) artery and the veins on the same view [7]. The radial nerve can be found just posterior to the axillary artery. Two branches of the brachial plexus are placed far from the axillary vessel (Figure 10).

**Figure 11:**
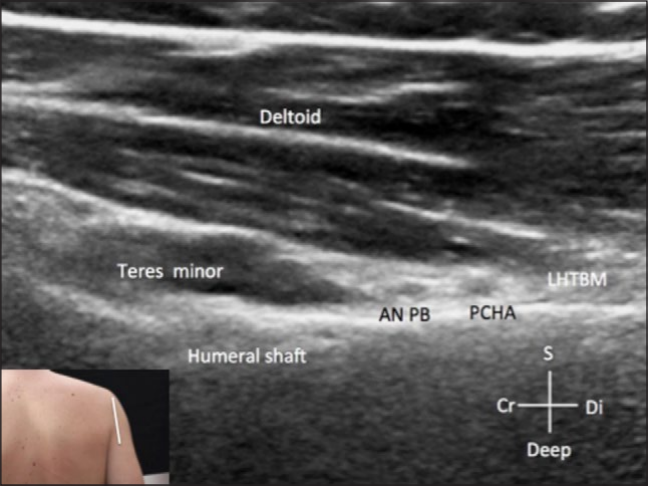
**Branches of the brachial plexus – axillary nerve – posterior approach.** ANPB: axillary nerve – posterior branch. Cr: cranial. Di: distal. S: superficial. LHTBM: lateral head of the triceps brachii muscle. PCHA: posterior circumflex humeral artery. The probe position is indicated by the white bar in the insert.

The musculocutaneous nerve in most patients is embedded between the brachial and the coracobrachial muscles medially to the other branches (Figure 10). The axillary nerve, which is the branch originating from the posterior cord, along with the radial nerve can be seen posterior to the humeral neck with the probe aligned along the long axis of the humerus and passing between the teres minor situated superiorly and the posterior circumflex artery and the lateral head of the triceps muscle located inferiorly [2]. This anatomic area is the well-known Velpeau’s quadrilateral space (Figure 11).

## Conclusion

US examination of the brachial plexus is a valuable alternative to MRI in cases of trauma, tumour involvement and fibrosis induced by a radiation oncology treatment.

US can also be used to guide interventional procedures for nervous blocks.

US of the brachial plexus requires a detailed knowledge of the anatomy and of the US standard views.

The main shortcomings of US examination of the brachial plexus are the lack of visibility of the spinal nerve roots, deeply located in the neuro-foramina, and a suboptimal demonstration of the C8 and T1 rami in some patients, especially those with a short neck and in children.
